# Determining Physiological and Energetic Demands during High-Level Pommel Horse Routines Using a Modified Method Based on Heart Rate–Oxygen Uptake Functions

**DOI:** 10.3390/sports12010027

**Published:** 2024-01-10

**Authors:** Alexander Seemann-Sinn, Peter Rüdrich, Tom Gorges, Ingo Sandau, Falk Naundorf, Bernd Wolfarth

**Affiliations:** 1 Department of Sports Medicine, Humboldt University of Berlin, 13353 Berlin, Germany; bernd.wolfarth@charite.de; 2 Department of Strength, Power and Technical Sports, Institute for Applied Training Science, 04109 Leipzig, Germany; gorges@iat.uni-leipzig.de (T.G.); sandau@iat.uni-leipzig.de (I.S.); naundorf@iat.uni-leipzig.de (F.N.); 3 Department of Sports Medicine, Institute for Applied Training Science, 04109 Leipzig, Germany; ruedrich@iat.uni-leipzig.de; 4 Department of Sports Medicine, Charité University of Medicine, 13353 Berlin, Germany

**Keywords:** artistic gymnastics, pommel horse, metabolic profile, aerobic, anaerobic

## Abstract

This study aimed (1) to assess the validity of a modified method (M_mod_) based on heart rate (HR)—oxygen uptake (VO_2_) regression functions to calculate total energy costs (W_total_) and aerobic (W_aer_) and anaerobic alactic energy contribution (W_pcr_) and (2) to analyse the physiological and energetic demands of high-level pommel horse routines (PH routines). The M_mod_ was developed because VO_2_ measurements are limited during high-level PH routines. Answering Part 1, nine male artistic gymnasts performed a PH routine where energy costs were calculated from VO_2_ measurements and then compared with energy costs determined from the HR- VO_2_ regressions of M_mod_’s two additional tests. Using the concordance correlation coefficient (CCC) and Deming regression, W_aer_ (CCC = 0.955), W_pcr_ (CCC = 0.999), and W_total_ (CCC = 0.990) show substantial to almost perfect validity without constant or proportional bias. Data from eight further gymnasts performing a high-level PH routine and a graded exercise test (GXT), as well as four data sets from Part 1, were used to determine physiological and energetic demands using M_mod_. VO_2_ and HR during PH routines reached 86.1% and 90.4% of the maximal values during GXT. W_pcr_ was 47.0%, anaerobic lactic energy contribution (W_blc_) was 29.7%, and W_aer_ was 23.3% of W_total_ required during PH routines. Summarising the energetic demands of high-level PH routines, they are mainly anaerobic, where W_pcr_ provides the largest energy share. W_aer_ provides a substantial part of W_total_ and should therefore also be specifically trained.

## 1. Introduction

Artistic gymnastics is listed as one of the most spectacular Olympic sports, as the artistry and skills performed by gymnasts often appear to be close to the ultimate limits of the human body [1]. To perform these skills at the highest technical level, gymnasts need different physical and psychological skills. In general, strength, speed, agility, and muscular endurance are considered essential physical skills in men’s artistic gymnastics (MAG) [2]. For example, gymnasts can enhance their muscular endurance by increasing metabolic efficiency and effectiveness via training specific metabolic pathways for energy production [2]. However, in MAG, knowledge about the relevance of the individual metabolic pathways is limited.

So far, for the pommel horse (PH), vault, parallel bars, and high bar, only calculations of energetic demands are present based on routine duration and blood lactate concentration (BLC). However, these calculations refer to outdated routine duration values [3] and therefore probably no longer represent the current energy demands on these apparatuses. On the other hand, for floor and still rings, there are studies that model energy supply using the PCr-LA-O_2_ method [4,5]. These studies show that in MAG, anaerobic energy supply is mainly realised by high-energy phosphates, and aerobic metabolism is an important energy source [4,5]. This is also confirmed in other acyclic activity sports, such as judo, indoor climbing, or rhythmic gymnastics, which involve a large number of short, high-intensity movements interspersed with short periods of less intense demands [6,7,8]. It should also be mentioned that in the MAG, only the study of still rings was performed with elite gymnasts and difficult routines. Of note, simpler routines do not represent the energetic demands of high-level routines and are therefore only of limited use for training recommendations at the top level.

PH, as one of the six apparatuses in MAG, is characterised by balance and rotation movements (circles), which are performed in side or cross support with closed or straddle legs in a continuous dynamic without interruption [9]. According to the international scoring regulations (Code de Pointage), PH routines must contain single-leg swings and scissors, circles and flairs with and/or without spindles and handstands, sweeps, Russian swings, flops, combined elements, travel-type elements, and a dismount to receive the full score [10]. Due to the frequent balance movements, the use of the PCr-LA-O_2_ method on the PH is limited since the required mobile spiroergometry system can lead to visual restrictions and balance irritations [11]. However, the relevance of modelling the energy requirements at PH is already given by the significant change in the routine duration [3], as routine duration substantially influences these requirements [12]. Indeed, the routine duration at PH increased by 22% after the fundamental modification of the Code de Pointage in 2005 [3]. There are also increases in physiological demands. For example, peak blood lactate concentrations (BLC_peak_) in PH routines increased from 6.00 to 8.70 mmol·L^−1^, while peak heart rate (HR_peak_) showed no increases [13]. In addition, measured data on peak oxygen consumption (VO_2peak_) for PH routines does not yet exist.

According to the previously mentioned estimates of energetic demand, energy supply during PH routines occurs after an initial contribution of the anaerobic-alactic system, mainly through the anaerobic-lactic system [14]. In contrast, a pilot study with three subjects using the PCr-LA-O_2_ method suggested that not the anaerobic-lactic system but the anaerobic-alactic and aerobic systems are the predominant energy suppliers during PH routines [11]. However, the use of the PCr-LA-O_2_ method with top athletes is not possible without modification, as this method requires a lot of training time with mobile spiroergometry, which is not feasible in professional sports.

Heart rate (HR), as an easily measured and accurate physiological parameter with no interfering factors for athletes, provides a way to calculate oxygen uptake (VO_2_) consumption via the HR- VO_2_ relationship [15]. In intermittent sports like tennis, basketball, or football, HR- VO_2_ relationship functions are widely used to calculate VO_2_ based on HR [15,16,17]. In this context, it should be noted that the HR- VO_2_ relationship varies from person to person, so it is necessary to determine the relationships between HR and VO_2_ individually [18]. Furthermore, it has been shown that the VO_2_ and energy costs determined by linear HR- VO_2_ relationships from ergometer-grade exercise tests overestimate the actual VO_2_ and energy costs [19]. Therefore, for acyclic exercise, a sport-specific adaptation of the HR- VO_2_ relationship functions generated on the ergometer is necessary. In this context, a study from tennis has shown that a sport-specific determination of the HR- VO_2_ relationship provides more accurate VO_2_ values than a semi-specific determination [15].

The first aim of the study was therefore to assess the concurrent validity of a modified method (M_mod_) to calculate total energy costs and relative energy contributions of metabolic pathways based on HR- VO_2_ relationship functions in PH. Based on the theoretical positions, the first hypothesis (H1) of this study was that in PH routines, the aerobic, anaerobic, alactic, and total energy costs can be validly calculated using M_mod_. The second aim of the study was to examine the physiological demands (VO_2_, BLC, and HR) and to determine the metabolic profile of elite PH routines using M_mod_. Based on the results of a pilot study and the duration of PH routines [3,11], the second hypothesis (H2) was that anaerobic alactic energy contribution (W_pcr_) provides the largest energy contribution in PH routines and that aerobic energy contribution (W_aer_) provides between 30% and 40% of the total energy. Finally, it would also be analysed whether there are any correlations between the energetic demands and the load characteristics of the PH routines (duration and difficulty) as well as between the energetic demands and the endurance performance (maximum VO_2_ [VO_2max_] and individual anaerobic threshold [IAT]) of the athletes. In this context, it was hypothesised (H3) that the endurance performance of the athletes correlates with the anaerobic lactic energy contribution (W_blc_).

## 2. Materials and Methods

### 2.1. Experimental Approach

The study was structured into two parts: (1) Validation of the modified method (M_mod_) for calculating total energy costs and relative energy contributions of metabolic pathways during PH routines; and (2) modelling of physiological and energetic demands during high-level PH routines in elite artistic gymnasts. Parts 1 and 2 of the study were basically conducted with different subjects, and due to the high performance level, data from 4 subjects from Part 1 was also included in Part 2 of the study. To test the validity of the M_mod_, the athletes in Part 1 of the study also completed the PH routine with mobile spiroergometry (original method [M_org_]). This procedure was very time-consuming because the athletes had to train for a long time with the mobile spiroergometry, which is therefore not practicable in professional sports. In Part 2, only the M_mod_ was used.

### 2.2. Subjects

Nine artistic gymnasts (age = 26.4 ± 5.0 years, height = 173.8 ± 5.4 cm, and weight = 68.2 ± 3.3 kg) participated in Part 1 of the study, and individual data are reported in Table 1. At the time of the study, all athletes performed as gymnasts in the German Gymnastics League. For Part 2 of the study, data from 8 gymnasts of the German national team as well as data from 4 subjects from Part 1 of the study were analysed, and individual data are also reported in Table 1. On average, the twelve gymnasts from Part 2 were 23.5 ± 4.23 years old, 174.0 ± 5.71 cm high, and weighed 66.5 ± 4.01 kg.

All athletes were free from injuries or other medical problems and were informed about the study procedure, benefits, and possible risks prior to the study. Before the start of the study, all athletes signed an informed consent. The study was approved by the Ethics Committee of the Faculty of Cultural, Social and Educational Sciences of the Humboldt University of Berlin (HU-KSBF-EK_2019_0017) and conducted in accordance with the Declaration of Helsinki.

### 2.3. Part 1: Validation of the Modified Method

#### 2.3.1. Procedure and Physiological Measurements of the Modified Method

**Figure 1:**
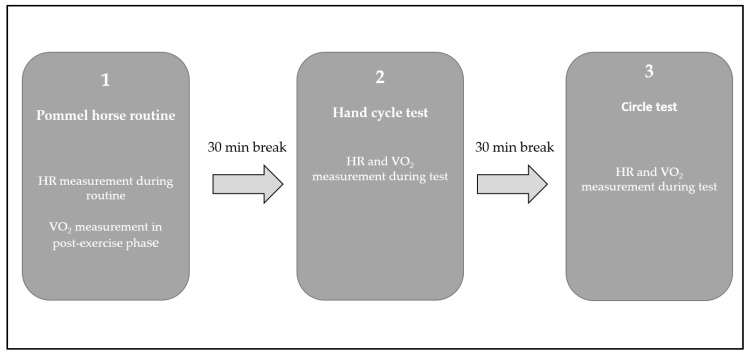
Experimental study design of the modified method.

The M_mod_ was developed for modelling W_aer_ and W_pcr_ during difficult PH routines. The M_mod_ consists of a PH routine and two additional performance tests (non-specific hand cycle test [HCT] and PH-specific circle test [CT]) to determine the athletes’ individual HR- VO_2_ relationship functions. Since determined HR- VO_2_ relationships on ergometers (treadmill, hand cycle, etc.) overestimate the actual VO_2_ [20], a PH-specific adaptation was made using the CT. In the CT, the subjects had to perform continuous circles on the PH over a certain period of time. The load in this test was below the load of a PH routine, so the mean value of the HR- VO_2_ functions from the HCT and CT was used to calculate the VO_2_ values of the PH routine. The M_mod_ was successfully tested on three subjects in an initial pilot study [11] and checked for validity in the present study. Figure 1 illustrates the experimental protocol design. As a first step in the M_mod_, the athletes completed an individual PH routine. During the PH routine, HR was measured with a HR sensor (HRM, Garmin Ltd., Schaffhausen, Switzerland) and VO_2_ was measured in the 10 min post-exercise phase using mobile spiroergometry (K5 Cosmed, Rome, Italy). The second test was the HCT, with a load duration between 40 and 50 s (depending on the duration of the corresponding PH routine of the athletes). The test started (like the CT) from rest, with the intensity of the load being 3 Watt·kg^−1^ bodyweight and a cranking frequency between 120 and 130 rpm. In a third step, the CT (same load duration as the HCT) was performed on the PH. During the HCT and CT, VO_2_ and HR were measured continuously breath-by-breath using mobile spiroergometry and a paired HR sensor. To avoid day-to-day variability of the HR- VO_2_ functions, the three tests were performed on one day. Between the tests, there was a 30 min break during which the subjects recovered passively.

#### 2.3.2. Calculation of VO_2_ during PH Routine

To improve the underlying characteristics of the VO_2_ measurement, obvious outliers of the VO_2_ values of the HCT and CT were manually eliminated. To determine the relationship between HR and VO_2_ values for HCT and CT, two exponential functions (Equations (1) and (2)) were fit into the data using non-linear least squares:

(1) $${{\mathrm{VO}}_{2}}_{\mathrm{HCT}}[\mathrm{mL}\times \min^{-1}]=a_{1}\;\times {\;e}^{b_{1}\;\times \;\mathrm{HR}\_\mathrm{HCT}}$$

(2) $${{\mathrm{VO}}_{2}}_{\mathrm{CT}}[\mathrm{mL}\times \min^{-1}]={\;a}_{1}\;\times \;e^{b_{1}\;\times \;\mathrm{HR}\_\mathrm{CT}}$$

To calculate the final VO_2_ values of the PH routine, the average of the calculated VO_2_ values of Equations (1) and (2) was used with the HR of the PH routine:

(3) $${{\mathrm{VO}}_{2}}_{\mathrm{PH}}[\mathrm{mL}\times \min^{-1}]=0.5({a_{1}\times {\;e}^{b_{1}\times \mathrm{HR}\_\mathrm{PH}\;\mathrm{routine}}+a}_{2}\;\times {\;e}^{b_{2}\times \mathrm{HR}\_\mathrm{PH}\;\mathrm{routine}\;})$$

The VO_2_ in the post-exercise phase of the PH routine was recorded using a mobile spiroergometry. After the end of the PH routine, the athletes walked a short distance (walking phase) to sit down on a chair, which lasted 10 s on average. As it took up to 15 s (walking phase + preparing mobile spiroergometry for measuring) until the mobile spiroergometry was ready to measure after the PH routine, no VO_2_ values could be recorded in the first 15 s of the post-exercise phase. Therefore, these missing VO_2_ values were interpolated using the cubic spline method. The walking phase was considered in the calculation of the energy shares due to the different physiological conditions [5]. Finally, using the calculated VO_2_ values, the physiological and energetic demands of the PH routine were modelled with the PCr-LA-O_2_ method established by Beneke et al. [21].

### 2.4. Part 2: Determination of Physiological and Energetic Demands for High-Level Pommel Horse Routines

#### 2.4.1. Graded Exercise Test

On two different days, the athletes performed a maximal graded exercise test (GXT) on a treadmill (Mercury T170 h/p/cosmos, Naussdorf-Traunstein, Germany) and the tests of the M_mod_ described above. The GTX was used to determine the maximum physiological parameters (HR_max_, VO_2max_, and BLCmax) as well as the IAT. Due to time problems, the GXT could only be performed with 9 of the 12 athletes.

On the GXT and M_mod_, breath-by-breath respiratory gas exchange and HR were recorded with a portable telemetric spiroergometry system (K5 Cosmed, Rome, Italy) coupled with an HR sensor (HRM, Garmin Ltd., Schaffhausen, Switzerland). The used K5 spiroergometry system shows excellent validity (R^2^ > 0.99) and reliability (intraclass correlation coefficient > 0.99) in breath-by-breath mode [22]. Prior to all tests, the spiroergometric system was calibrated using ambient room air, reference gas (5.0% CO_2_ and 16.0% O_2_), and a defined 3 L air volume according to the manufacturer’s instructions.

The initial speed of the GXT was 6.0 km/h and was increased by 2.0 km/h every 3 min. The treadmill incline was set at 1%. Once the subjects felt subjectively exhausted (perceived level of exertion between 19 and 20 using the 6 to 20 Borg scale [23], the test was stopped). VO_2_ and HR were measured continuously during the test. HR_max_ was defined as the mean value over the last 10 s of exercise, and VO_2max_ as the mean value over the last 30 s of exercise. IAT was determined according to the Dickhuth method [24].

#### 2.4.2. Procedure and Physiological Measurements for Determination of Physiological and Energetic Demands

Each subject performed an individual PH routine as described in the M_mod_, followed by the two performance tests of the M_mod_ (HCT and CT). For the exact determination of the start and end of the routine as well as the difficulty score of the routine (D-score), the routine (including the pre- and post-load phases) was recorded with a video camera (GC-PX100 JVC, Yokohama, Japan). The D-score of the PH routine was determined according to the Code de Pointage of the International Gymnastics Federation [10]. The execution of the routine was not judged, but the athletes had to perform their routine in good quality. Prior to the start of the PH routine, the subjects had adequate time to practice. A cool-down phase followed the practice to restore the resting level of the physiological parameters. After the cool-down phase, the two-minute prestart phase began, during which prestart lactate was taken and HR measurement and video recording were started. After the end of the PH routines, the subjects walked a short distance (walking phase) to sit down on a chair, which lasted 10 s on average. After the walking phase and preparing the mobile spiroergometry, the VO_2_ measurement started, which was performed until 10 min after the tests. HR was measured continuously during the 2 min pre-start phase, during the PH routines, and also until 10 min after the end of the test. By using equation 3 of the M_mod_ and the interpolation of the missing 15 s of post-exercise values, the VO_2_ data were calculated. Finally, the VO_2_ post-exercise values recorded by spiroergometry (breath-by-breath) were interpolated to 1-s values using the cubic spline method (Origin, 2021).

HR_peak_ and VO_2peak_ have been calculated by averaging the last 5 s of the PH routines. BLC was analysed using 20 μL of capillary blood from the hyperaemic earlobe. The blood was taken in the pre-start phase, after the end of the routine, and every minute up to the eighth minute of the post-exercise phase using end-to-end glass capillaries, and finally analysed using a blood analyser (SuperGl, Dr. Müller Gerätebau, Freital, Germany). One minute after the PH routine, the athletes were asked to rate their RPE on the 6 to 20 Borg scale.

### 2.5. Calculation of Energy Contributions

The calculation of the energy contributions of W_aer_, W_blc_, and W_pcr_ was conducted for parts 1 (validation) and 2 (physiological energetic requirements) of the study using the PCr-LA-O_2_ method [21]. W_aer_ was calculated from the VO_2_ above the resting metabolic rate and caloric equivalent [25] by using:

(4) $$W_{\mathrm{aer}}\;[\mathrm{kJ}]={VO}_{2}(\mathrm{mL})\;\times \;\mathrm{caloric}\;\mathrm{equivalent}\;(J\times {mL}^{-1})\;\times {\;1000}^{-1}$$

VO_2_ above resting metabolic rate was determined as the area under the curve of actual VO_2_ minus resting metabolic rate. Resting metabolic rate was calculated using the Bendict-Harris formula [26]. The required caloric equivalent was defined as 20.9 J·mL^−1^ [12]. W_blc_ was calculated from the highest change in BLC (ΔBLC), the oxygen lactate equivalent of 3.0 mL·O_2_·kg^−1^·mmol^−1^·L^−1^ [25], and body weight using:

(5) $$W_{\mathrm{blc}}\;[\mathrm{kJ}]=\Delta \mathrm{BLC}\;\times \;3.0\;(\mathrm{mL}\times \;O_{2}\times \;{{\mathrm{kg}}^{-1}\times L}^{-1})\;\times \;\mathrm{caloric}\;\mathrm{equivalent}\;(J\times {\mathrm{mL}}^{-1})\;\times \;1000^{-1}$$

W_pcr_ was considered the fast component of post-exercise VO_2_ and was calculated as follows:

(6) $$W_{\mathrm{pcr}}\;[\mathrm{kJ}]={VO}_{2\mathrm{pcr}}(\mathrm{mL})\;\times \;\mathrm{caloric}\;\mathrm{equivalent}\;(J\times {\mathrm{mL}}^{-1})\;\times \;1000^{-1}$$

To account for the walking phase, the VO_2_ post-exercise curve was split into 2 phases, and the VO_2_ of the fast component was calculated according to the procedure described in [5]. The split time was defined as the end of the walking phase and determined manually from the video recordings. Besides the physiological background, the split of the postexercise VO_2_ improved the quality of the curve fitting (reduction of the average residual standard error by 7.1%).

Finally, the total energy (W_total_) was calculated as the sum of the contributions of the individual energy systems:

(7) $$W_{\mathrm{total}}\;[\mathrm{kJ}]=W_{\mathrm{aer}}+W_{\mathrm{blc}}+W_{\mathrm{pcr}}$$

And metabolic power was calculated as W_aer_, W_blc_, W_pcr_, and W_total_ divided by routine duration. All energy shares were calculated in kJ and are presented in absolute (kJ) and relative (% of W_total_) numbers.

### 2.6. Statistical Analysis

Statistical analyses were conducted using statistical software products (Excel 2016, Microsoft, Redmond, WA, USA, and Jamovi [Version 2.3]). For Part 1 of the study, descriptive statistics were performed, and the data were presented as mean ± standard deviation (SD). To check validity, the normal distribution of the differences (M_org_ vs. M_mod_) was tested and confirmed using the Shapiro-Wilk test. Relative validity was tested using Lin’s concordance correlation coefficient (CCC) with 95% confidence intervals (CI). Before using the CCC, the other criteria, heteroscedasticity and proportional bias, were tested and not confirmed. Therefore, no log-transformation of the raw data was necessary. The agreement criteria for the CCC were classified as poor (CCC < 0.90), moderate (CCC < 0.95), substantial (CCC < 0.99), and almost perfect (CCC ≥ 0.99) [27]. For the assessment of absolute validity, first a Deming regression was performed to check for constant and proportional bias between the methods. Deming regression was used because the determination of energy costs using mobile spiroergometry can also be affected by measurement errors, and Deming regression is a method for estimating the relationship between two sets of measurements, taking into account measurement error in both variables. In the absence of constant and proportional bias between the methods, a Bland-Altman analysis was performed to calculate the 95% limits of agreement (LoA) and the SD of measurement differences (SDD; random measurement error). The SDD was used to quantify the error in calculated W_aer_, W_pcr_, and W_total_ using M_mod_.

For Part 2 of the study, descriptive statistics were also performed, and data were presented as mean ± SD and 95% CI. Pearson r was used to analyse statistical correlations between the parameters.

## 3. Results

The results are divided into two parts, with Part 1 presenting the results of the validation of the M_mod_ and Part 2 presenting the physiological and energetic demands of the high-level PH routines.

### 3.1. Part 1: Validity of the Modified Method

Table 2 shows the descriptive data for W_aer_, W_pcr_, and W_total_ using M_org_ and M_mod_.

W_pcr_ presents almost perfect relative validity, while W_aer_ and W_total_ have substantial relative validity (Table 3). For all three parameters, there are no significant proportional or constant biases.

**Figure 2:**
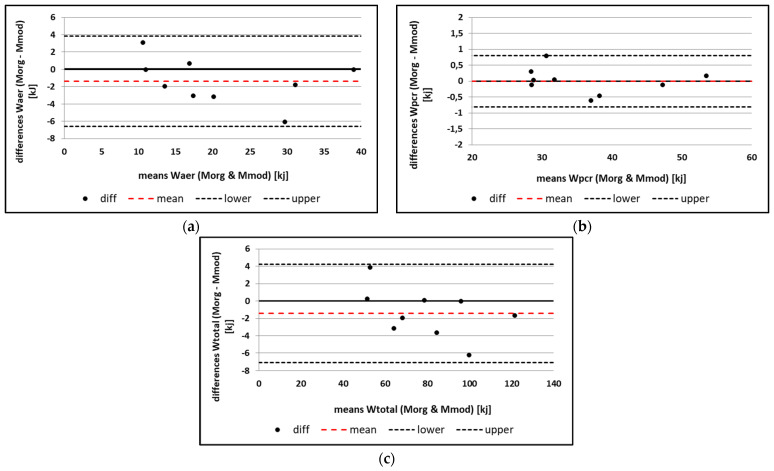
Bland-Altman analysis for the comparison of (**a**) W_aer_; (**b**) W_pcr_, and (**c**) W_total_ measured using M_org_ and M_mod_ (black dashed lines, upper and lower limits of agreement; red dashed line, mean difference between M_org_ and M_mod_).

From the Bland-Altman analysis, the mean difference for W_aer_ between M_org_ and M_mod_ is −1.396 kJ, with LoA ranging from −6.611 kJ to 3.819 kJ (Figure 2). The SDD is 2.661 kJ (12.63%). For W_pcr_, the mean difference is −0.003 kJ with a LoA ranging from −0.811 kJ to 0.806 kJ (Figure 2). The SDD is 0.412 kJ (1.14%). W_total_ shows a mean difference of −1.398 kJ with LoA ranging from −7.047 kJ to 4.251 kJ (Figure 2). The SDD is 2.882 kJ (3.61%).

**Figure 3:**
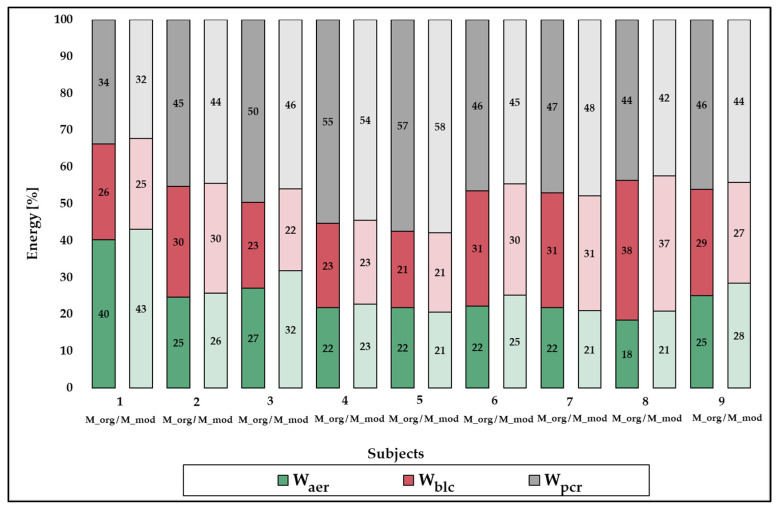
Individual relative contributions of energy supply during the pommel horse routines of the nine subjects from Part 1 using M_org_ (dark colours) and M_mod_ (light colours). W_aer_ indicates energetic contributions from the aerobic energy system; W_blcC_, energetic contributions from the anaerobic lactic system; W_pcr_, energetic contributions from the anaerobic alactic system.

Looking at the individual values of the relative energy contributions measured with M_org_ and M_mod_ shows that there are no major differences in the distribution of energy supply (Figure 3). There is a maximum deviation of 5%, whereby the general tendency of the energetic requirements of PH routines remains the same for all tested athletes.

### 3.2. Part 2: Physiological and Energetic Data of High-Level Pommel Horse Routines

The subjects reached a maximal VO_2_ of 52.5 ± 4.4 mL·min^−1^·kg^−1^, a maximal HR of 189.6 ± 7.3 beats per minute, a maximal blood lactate concentration of 8.8 ± 2.0 mmol·L^−1^, and an IAT of 11.6. ± 1.3 km/h during the GXT. Table 4 shows the performance and physiological data of the PH routines. Data shows that lower VO_2peak_ and HR_peak_ values, but higher BLC_peak_ values, were reached during the PH routines than during GXT. VO_2peak_ and HR_peak_ of the PH routine are 86.1 ± 12.2% and 90.4 ± 4.8% of the values from the GXT. BLC_peak_ values reach 90.2 ± 17.4%.

**Figure 4:**
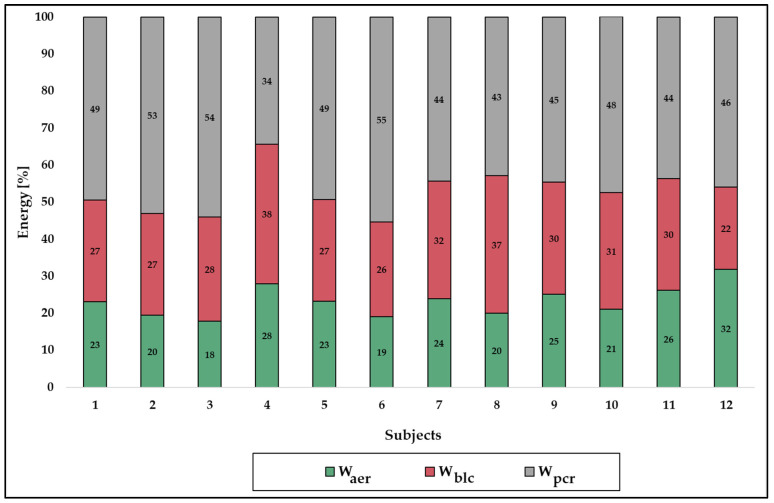
Individual relative contributions of energy supply during the pommel horse routines of the twelve subjects from Part 2. W_aer_ indicates energetic contributions from the aerobic energy system; W_blc_, energetic contributions from the anaerobic lactic system; W_pcr_, energetic contributions from the anaerobic alactic system.

The modelled energy demands of the PH routines as well as the relative energy contributions and metabolic power are shown in Table 5. Figure 4 displays the relative contribution of the energy systems according to each athlete. On the basis of the averaged residual standard error, the goodness of fit for the curve fitting process (R^2^) for the VO_2_ post-exercise curve was 0.93 (±0.02).

**Figure 5:**
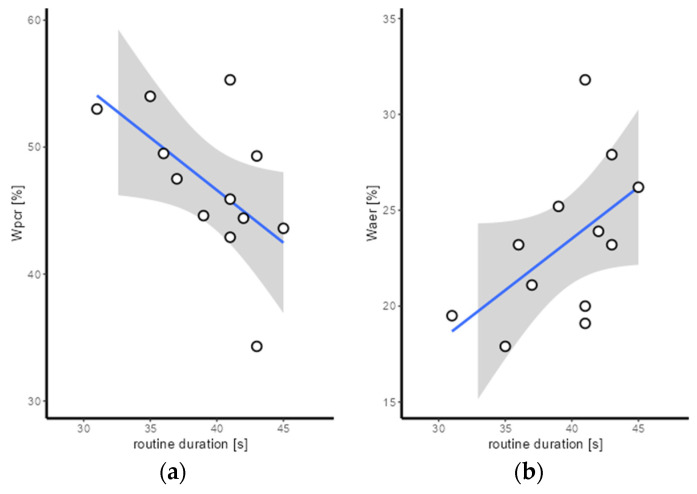

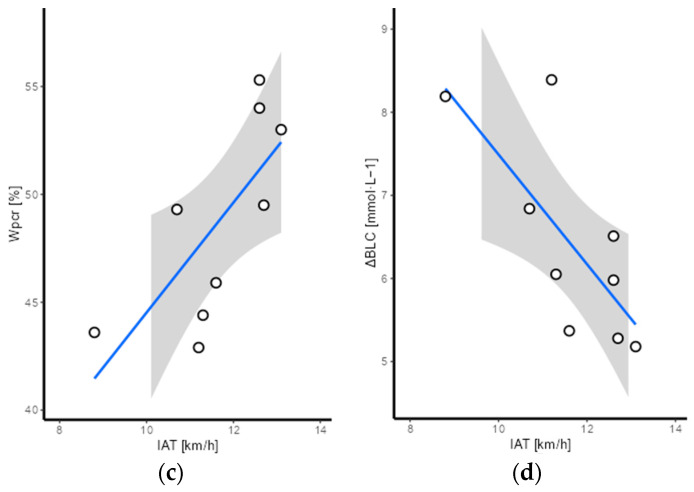
Scatter plot for the comparison of (**a**) routine duration and relative W_aer_; (**b**) routine duration and relative W_pcr_; (**c**) IAT and relative W_pcr_; and (**d**) IAT and ΔBLC (blue lines, regression line; grey surface, confidence interval on regression line).

In terms of correlations, there is a significant negative correlation between routine duration and relative W_pcr_ (r = −0.577; *p* = 0.05) (Figure 5). No further significant correlations were found between the performance data of the PH routines (duration and difficulty) and the relative energy contributions. However, there is a positive trend between routine duration and relative W_aer_ (r = 0.532; *p* = 0.075) (Figure 5). Moreover, there is a significant positive correlation between IAT and relative W_pcr_ (r = 0.726; *p* = 0.027) and a significant negative correlation between IAT and ΔBLC (r = −0.738; *p* = 0.023) (Figure 5). Further significant correlations between physiological data from GXT and the relative energy contributions have not been found.

## 4. Discussion

The aim of the present study was (1) to assess the concurrent validity of a M_mod_ based on individual HR- VO_2_ functions to determine the energy cost during high-level PH routines and (2) to model the physiological and energetic demands of high-level PH routines in elite artistic gymnasts using the newly developed M_mod_. Consistent with our hypothesis (H1), the results show substantial to almost perfect validity for W_aer_ (CCC = 0.955), W_pcr_ (CCC = 0.999), and W_total_ (CCC = 0.990) using M_mod_. All three parameters did not show any constant or proportional bias. These results are in line with those of Baiget, Iglesias, and Rodriguez [15] and Scribbans, Berg, Narazaki, Janssen, and Gurd [16], who also demonstrated that energy costs and VO_2_ values can be determined sufficiently validly for intermittent acyclic exercise using HR- VO_2_ relationship functions. Earlier studies regarding energy costs on PH, which were also based on individual HR- VO_2_ relationships, did not compare the results with direct VO_2_ measurements during the routines [28]. Therefore, no statement on the validity of such a method on PH could be made so far. Also, no PH-specific adaptation of the HR- VO_2_ relationship function was carried out in the earlier studies. In the present study, the random error of W_aer_ is 12.6%, that of W_pcr_ is 1.1%, and that of W_total_ is 3.6%. One possible reason for this error could be differences in the VO_2_ and HR kinetics, since VO_2_ recovers faster and more completely than HR during periods of low intensity [19]. Another error could be the emotional stress prior to the routines, which increases HR above metabolic requirements at the onset of the routine (HR sympathetic drive) [15]. The problem of increased HR can be seen in subject 3 in Figure 2 (also the outlier in the Bland Altman plots) and leads to an increase in the random errors of M_mod_. Removing this athlete from the analysis would result in a random error of 10.6% for W_aer_, 1.3% for W_pcr_, and 3.1% for W_total_. For future studies, one way to minimise this source of error would be to define a maximum pre-start HR value for each athlete based on individual HR reference values. Despite the aforementioned limitations, the M_mod_ offers the possibility of modelling the energetic demands, including the calculation of the energy costs of the individual metabolic pathways as well as the modelling of the physiological demands during high-level PH routines. This is shown in Figure 2, where no major difference in the relative energy contributions between the M_org_ and M_mod_ can be observed. Modelling the energetic and physiological demands of high-level PH routines has, to our knowledge, not been performed so far and thus provides further important information for gymnastics-specific muscular endurance performance.

Looking at the performance and physiological data of the PH routine studied, the achieved HR_peak_ values are slightly higher than those of French elite gymnasts, while the BLC_peak_ values are at similar levels [13]. Comparing the values with those of other apparatus or similar sports shows that the HR_peak_ values are similar to those on still rings (172 bpm) or indoor climbing (181 bpm), while the BLC_peak_ values are higher (still rings 6.1 mmol·L^−1^; indoor climbing 3.8 mmol·L^−1^) [5,7].This could be due to the more cyclic movement pattern of the PH routines. The VO_2peak_ values modelled for the first time also correspond to those of still-ring routines but are higher than those for indoor climbing [5,7]. The achieved VO_2peak_ and HR_peak_ correspond to 86.1 ± 12.2% and 90.4 ± 4.8% of the values from the GXT and confirm the intensive stress on the cardiopulmonary system in MAG [5,13].

The modelled energetic demands show that W_pcr_ provides 47.0%, W_blc_ 29.7%, and W_aer_ 23.3% of the total energy required for high-level PH routines. Thus, the results confirm the hypothesis (H2) that W_pcr_ provides the largest energy contribution and contradict previous assumptions of a dominant anaerobic lactic metabolism on PH. The increase in peak power observed in gymnasts over the last four decades [29] may have influenced this result. Peak power and anaerobic alactic capacity seem to be key components of the physiological profile of elite gymnasts [5,29]. However, when W_pcr_ and W_blc_ are combined, PH routines are mainly anaerobic, which is in line with previous assumptions [14]. This anaerobic dominance could also be confirmed on still rings [5]. With 23.3%, the hypothesis of a 30–40% relative energy supply by W_aer_, which would have been expected due to the routine duration [12], cannot be confirmed. This is in line with results on still rings and indoor climbing, where the modelled relative W_aer_ was also slightly lower than the expected W_aer_ [5,7]. It seems that in mainly upper-body short-duration acyclic sports with highly explosive movements and high technical focus, routine duration has limited validity as a predictor of relative W_aer_. As the upper limbs have a greater proportion of type II fibres [30] and slower VO_2_ kinetics than the lower limbs [31], the main use of upper limb muscles could explain the lower use of the aerobic system. Nevertheless, with 23.3%, W_aer_ is not insignificant and should be trained specifically. Especially since the aerobic energy supply on PH increases with increasing routine duration [14]. This is also indicated by the positive trend between routine duration and relative W_aer_ observed in this study. Improved aerobic power could, for example, reduce high lactate accumulation and associated muscular fatigue during the PH routine. This fact has also been shown in still rings or ballet routines, where an increase in aerobic power reduces relative W_blc_ [5,32]. In this study, there was no significant correlation between aerobic power and relative W_blc_, so the hypothesis (H3) cannot be confirmed. Only a moderate negative trend between IAT and relative W_blc_ was observed. However, there was a significant negative relationship between IAS and ΔBLC. This suggests that improved aerobic power can reduce lactate accumulation during PH routines.

The modelled metabolic profile and metabolic power of PH routines differ slightly compared with metabolic profiles on floors, still rings, or other short-duration acyclic sports. Compared with still rings and indoor climbing, relative W_blc_ is higher (still rings 20.5%; indoor climbing 13.8%), while relative W_pcr_ and W_aer_ are slightly lower (still rings 50.9% and 28.6%; indoor climbing 42.4% and 43.8%). This could be due to the slightly different movement structure and routine duration. PH routines have a more cyclic character and a shorter routine duration, which favours anaerobic lactic energy supply. Still rings routines and indoor climbing, on the other hand, are characterised by intermittent static movements and small breaks between elements or climbing moves, as well as a longer routine duration, which tends to favour anaerobic, alactic, and aerobic metabolism. On the floor, W_aer_ is the dominant energy source (54.4%), while W_blc_ (18.9%) provides the lowest energy share. The aforementioned slower VO_2_ kinetics of the upper body and the higher proportion of type II fibres, as well as the significantly shorter routine duration of the PH routine, could be responsible for these differences. Metabolic power is 34.80 ± 3.49 W · kg^−1^, higher than on still rings (30.7 ± 4.8 W · kg^−1^) or on floor (21.9 W · kg^−1^), but significantly lower than on a 30s Wingate test (58.0 ± 5.4 W · kg^−1^) [4]. This also shows that a shorter routine duration and a more cyclic movement profile can influence metabolic power. Further study on the metabolic profiles of other gymnastics apparatuses could provide more information. Moreover, the already-mentioned estimates of energetic demands based on routine duration and BLC could be verified.

When interpreting the results, however, the following limitations of the study must be taken into account. The experimental process was routine in a simulated competition and was carried out with a small sample size. Therefore, the available data should be compared with future studies. A further limitation is that, in addition to aerobic power (VO_2peak_), no anaerobic power of the athletes was measured. Differences in anaerobic power could influence the energy supply and be a reason for the individual differences in the energy demands of the athletes. In addition, no data were collected concerning the muscle and fat mass of the athletes, as this was not part of the general medical check-up of the athletes. Considering that a higher contracting muscle mass increases the total amount of ATP-PCr [33], the anaerobic alactic energy fraction could have been influenced by a different muscle mass. Therefore, due to the possible influence of muscle mass and the anaerobic power of the athletes, the general validity of the results should be considered with caution. On the other hand, this offers a perspective for further studies: How do anaerobic performance and muscle mass affect the energetic demands of high-level PH routines?

## 5. Conclusions

To our knowledge, this is the first study modelling the energetic and physiological demands of high-level PH routines using a M_mod_ based on HR- VO_2_ relationship functions. The M_mod_ was found to be a practicable and valid method for this purpose. The determined physiological demands show that high-level PH routines with 86.1 ± 12.2% of VO_2max_ and 90.4 ± 4.8% of HR_max_ place high demands on the aerobic and cardiovascular systems. The calculated metabolic profile differs from previous assumptions in the literature. The anaerobic alactic and, not as previously assumed, the anaerobic lactic metabolic pathways represent the dominant energy source during high-level PH routines. Thus, peak power as well as anaerobic alactic capacity seem to be key components of the physiological profile of elite gymnasts. The aerobic metabolism provides a substantial part of the total energy and should therefore also be specifically trained. Especially as its energy share seems to increase with increasing routine duration. The demonstrated positive correlation between IAT and ΔBLC shows that a higher aerobic power (and thus also a higher IAT) can lead to further benefits. A higher IAT could reduce excessive lactate accumulation and minimise the associated muscular fatigue during high-level PH routines. Future studies could examine whether an improved IAT also has a positive effect on the other apparatus and possibly identify the IAT as another key component of the physiological profile of elite gymnasts.

## Figures and Tables

**Table 1:** Individual demographic data of the athletes.

Part of the Study	Subjects	Age (Years)	Height (cm)	Body Mass (kg)	Years of Experience	Weekly Training Hours	VO_2peak_ (mL·kg^−1^·min^−1^)	IAS (km/h)
1	1	20	174	68	14	10–15	-	-
	2	27	177	72	23	20–25	-	-
	3	27	174	68	23	20–25	-	-
	4	35	160	62	31	15–20	-	-
	5	28	174	68	23	5–10	-	-
	6	32	174	67	28	15–20	-	-
	7	25	177	67	20	15–20	-	-
	8	20	178	74	14	10–15	-	-
	9	24	176	68	18	10–15	-	-
2	1	27	167	63.0	23	≥28	58.7	12.7
	2	18	179	62.0	14	≥28	59.0	13.1
	3	19	176	61.0	15	≥28	53.0	12.6
	4	20	178	66.0	15	≥28	-	-
	5	23	175	68.0	19	≥28	48.7	10.7
	6	22	160	61.0	17	≥28	55.6	12.6
	7	23	173	72.5	19	≥28	49.2	11.3
	8	19	181	70.0	15	≥28	52.6	11.2
	9 *	32	174	67.0	28	15–20	-	-
	10 *	25	177	67.0	20	15–20	-	-
	11 *	27	177	72.0	23	20–25	-	8.8
	12 *	27	174	68.0	23	20–25	49.0	12.1

Abbreviation: *, also subject in Part 1 of the study; VO_2peak_, peak oxygen uptake; IAS, individual anaerobic threshold.

**Table 2:** Descriptive data of W_aer_, W_pcr_, and W_total_ (mean ± SD) using M_org_ and M_mod_.

Parameters	M_org_	M_mod_
W_aer_ (kJ)	20.37 ± 9.58	21.77 ± 10.55
W_pcr_ (kJ)	36.06 ± 8.98	36.06 ± 9.07
W_total_ (kJ)	79.08 ± 22.65	80.48 ± 24.00

Abbreviations: W_aer_, energetic contributions from the aerobic energy system; W_pcr_, energetic contributions from the anaerobic alactic system; W_total_, total energy costs.

**Table 3:** Measures of validity of M_org_ and M_mod_.

Parameters	Intercept	Slope	SDD	lLOA	uLOA	CCC
	(95% CI)	(95% CI)		(95% CI)	95% CI	(95% CI)
W_aer_	0.669	0.905	2.661	−6.611	3.819	0.955
	(−5.736; 7.070)	(0.579; 1.230)		(−10.247; −2.974)	(0.183; 7.456)	(0.833; 0.988)
W_pcr_	0.339	0.991	0.412	−0.811	0.806	0.999
	(−1.369; 2.050)	(0.941; 1.040)		(−1.374; 0.247)	(0.242; 1.369)	(0.996; 1.000)
W_total_	3.162	0.943	2.882	−7.047	4.251	0.990
	(−6.795; 13.12)	(0.818; 1.070)		(−10.986; −3.108)	(0.312; 8.189)	(0.964; 0.997)

Abbreviations: SDD, standard deviation of differences (in kJ); lLOA, lower limits of agreement (in kJ); uLOA, upper limits of agreement (in kJ); CCC, concordance correlation coefficient; CI, confidence intervals; W_aer_, energetic contributions from the aerobic energy system; W_pcr_, energetic contributions from the anaerobic alactic system; W_total_, total energy costs.

**Table 4:** Performance and physiological data of the pommel horse routines.

Parameter	Mean ± SD	95% CI
routine duration (s)	39.50 ± 4.03	36.9–42.1
D-score (Ppoints)	4.72 ± 0.48	4.4–5.0
HR_mean_ (bpm)	149 ± 10.1	142–155
HR_peak_ (bpm)	172 ± 8.37	166–177
VO_2mean_ (mL·min^−1^·kg^−1^)	30.70 ± 5.39	27.3–34.1
VO_2peak_ (mL·min^−1^·kg^−1^)	45.4 ± 5.03	42.2–48.6
BLC_pre_ (mmol·L^−1^)	1.45 ± 0.82	0.93–1.97
BLC_post_ (mmol·L^−1^)	7.92 ± 1.25	7.12–8.71
ΔBLC, (mmol·L^−1^)	6.47 ± 1.11	5.77–7.17
Rating of perceived exertion	15.5 ± 1.27	14.6–16.4

Abbreviations: ΔBLC, highest change in blood lactate concentration from the pommel horse routines; BLC_peak_, peak blood lactate concentration from the pommel horse routines; BLC_pre_, blood lactate concentration before the pommel horse routines; D-score, difficulty score; HR_mean_, mean heart rate from the pommel horse routines; HR_peak_, peak heart rate from the pommel horse routines; VO_2mean_, mean oxygen uptake from the pommel horse routines; VO_2peak_, peak oxygen uptake from the pommel horse routines.

**Table 5:** Absolute energy, relative energy, and metabolic power of the energy systems of the pommel horse routines.

	Absolute Energy [kJ]		Relative Energy [%]		Metabolic Power [W·kg^−1^]	
	Mean ± SD	95% CI	Mean ± SD	95% CI	Mean ± SD	95% CI
W_aer_	21.40 ± 6.07	17.60–25.30	23.30 ± 4.08	20.70–25.80	8.05 ± 1.50	7.10–9.00
W_blc_	27.10 ± 5.68	23.50–30.70	29.70 ± 4.45	26.90–32.60	10.30 ± 1.22	9.48–11.00
W_pcr_	42.80 ± 7.65	37.90–47.60	47.00 ± 5.79	43.30–50.7	16.50 ± 3.31	14.40–18.60
W_total_	91.30 ± 14.50	82.10–101.00	100	-	34.80 ± 3.49	32.6–37.0

Abbreviations: SD, standard deviation; CI, confidence intervals; W_aer_, energetic contributions from the aerobic energy system; W_blc_, energetic contributions from the anaerobic lactic system; W_pcr_, energetic contributions from the anaerobic alactic system; W_TOTAL_, total energy costs.

## Data Availability

The data used to support the findings of this study are available from the corresponding author upon request. The data are not publicly available due to ethical restrictions.
